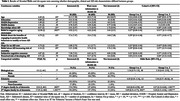# Demographic, clinical and Alzheimer’s disease risk characteristics associated with interest in *APOE* disclosure amongst middle‐aged Australian adults

**DOI:** 10.1002/alz.090534

**Published:** 2025-01-09

**Authors:** Emily Rosenich, Hannah Olver, Stephanie Perin, Ruby Haeusler, Tamar Foreman, Jason Karlawish, Yen Ying Lim

**Affiliations:** ^1^ Turner Institute for Brain and Mental Health, Monash University, Melbourne, VIC Australia; ^2^ University of Pennsylvania, Philadelphia, PA USA

## Abstract

**Background:**

Clinical guidelines in Australia discourage disclosure of apolipoprotein E (*APOE*) genotypes but advances in Alzheimer’s disease (AD) therapeutics will likely change this. Limited work has assessed interest in *APOE* disclosure in Australian adults, and it remains unclear which characteristics are associated with interest within this sample. In a large group of middle‐aged adults, this study aimed to describe interest in *APOE* disclosure and investigate differences in demographic and clinical characteristics and AD risk perceptions among groups with varying interest in disclosure.

**Method:**

Cognitively unimpaired middle‐aged adults aged 40‐70 (N = 460) enrolled in the Healthy Brain Project or BetterBrains Trial completed the Knowledge, Interest and Preferences for *APOE* Testing and Disclosure questionnaire. Online assessments measured demographics (age, sex, education, ethnicity, dementia family history), anxiety and depressive symptoms, subjective cognition, perceived risk of developing AD, and knowledge of AD. Participants were categorized into groups based on self‐reported interest in disclosure (interested/want more information/not interested). Frequencies described interest in *APOE* disclosure and perceived benefits and concerns. Kruskal‐Wallis and chi‐square tests assessed whether demographic and clinical characteristics and AD risk perceptions differed between groups.

**Result:**

Only 6% of participants were not interested, with most either interested (51%) or wanting more information about *APOE* (43%). Compared to participants who were not interested, interested participants were younger, more knowledgeable about AD, more likely to report any family history of dementia, had stronger beliefs in negative aging stereotypes and about the benefits of engaging in protective health behaviors to modify AD risk, had greater hope for an AD cure, and greater perceived risk of AD‐related mortality (Table 1). Amongst interested participants, motivation to change behaviors to reduce AD risk was the most common perceived benefit of disclosure (82%), whereas worry about attributing subtle memory changes to impending AD dementia was the most common concern (23%).

**Conclusion:**

Most middle‐aged Australian adults sampled expressed an interest in *APOE* disclosure in a research setting. Contrary to previous findings, greater subjective cognitive concerns did not influence interest. Instead, results highlight that an array of biopsychosocial factors influence interest in *APOE* disclosure. Findings will inform the development of disclosure protocols for Australian populations.